# Supplementary material for: Epilepsy detection based on multi-head self-attention mechanism
Source: PLoS One. 2024 Jun 11;19(6):e0305166. doi: 10.1371/journal.pone.0305166 (PMC11166279; doi:10.1371/journal.pone.0305166)
Supplement: Attachment — Submitted filename: Comments_PONE-D-24-07075.docx [file pone.0305166.s001.docx]

1. The motivation for research is not clear from the abstract.
2. Paper contributions should be considered in the introduction section.
3. Paper organization is missing in the introduction section.
4. The related work section is missing in the paper.
5. Please improve the figure quality.
6. Please consider a system overview or block diagram of research for a better understanding of the paper.
7. Data distribution graphs should be considered for the analysis.
8. Limitations of the proposed approach should be discussed.
9. In Figs 7 and 8, the y label should be written.
